# Case Report: A Case of Epstein-Barr Virus-Associated Acute Liver Failure Requiring Hematopoietic Cell Transplantation After Emergent Liver Transplantation

**DOI:** 10.3389/fimmu.2022.825806

**Published:** 2022-01-28

**Authors:** Koji Nakajima, Eitaro Hiejima, Hiroshi Nihira, Kentaro Kato, Yoshitaka Honda, Kazushi Izawa, Naoko Kawabata, Itaru Kato, Eri Ogawa, Mari Sonoda, Tatsuya Okamoto, Hideaki Okajima, Takahiro Yasumi, Junko Takita

**Affiliations:** ^1^ Department of Pediatrics, Kyoto University Hospital, Kyoto, Japan; ^2^ Department of Pediatric Surgery, Kyoto University Hospital, Kyoto, Japan; ^3^ Department of Pediatric Surgery, Kanazawa Medical University, Ishikawa, Japan

**Keywords:** hemophagocytic lymphohistiocytosis (HLH), Epstein-Barr virus (EBV), acute liver failure (ALF), hematopoietic cell transplantation (HCT), case report, CAEBV infection

## Abstract

Hepatic manifestations of Epstein-Barr virus (EBV) infection are relatively common, mild, and self-limiting. Although fulminant hepatic failure has been reported in a few cases, the contributing factors are unclear. This report discusses a pediatric case of EBV-associated acute liver failure that required urgent liver transplantation; however, liver damage continued to progress post-liver replacement. Monoclonal CD8+ T cells that preferentially infiltrated the native and transplanted liver were positive for EBV-encoded small RNA, suggesting a pathophysiology similar to that of EBV-associated hemophagocytic lymphohistiocytosis and chronic active EBV infection. Therefore, subsequent chemotherapy and hematopoietic cell transplantation was conducted, which led to cure. This is the first case of EBV-associated acute liver failure that relapsed post-liver transplant. As such, it sheds light on an under-recognized clinical entity: liver-restricted hyperinflammation caused by EBV-infected monoclonal CD8+ T cells. This phenomenon needs to be recognized and differentiated from hepatitis/hepatic failure caused by EBV-infected B cells, which has a relatively benign clinical course.

## Introduction

Epstein-Barr virus (EBV), a member of the herpes virus family, causes a self-limiting infectious mononucleosis. The pathogenesis of infectious mononucleosis is driven by T cells specific for EBV-infected B cells, which results in the triad of fever, pharyngitis, and lymphadenopathy, and often accompanied by mild liver enzyme elevations (1). Occasionally, EBV infects T cells and causes the disease spectrum referred to as EBV- associated T and NK- cell lymphoproliferative disease (EBV-LPD). The two representative forms of EBV-LPD are EBV-associated hemophagocytic lymphohistiocytosis (EBV-HLH) and chronic active EBV disease (CAEBV). Both are treated with chemotherapy or, if unresponsive, with hematopoietic cell transplantation (HCT).

Here, we describe a pediatric case that presented initially with presumed acute liver failure. Liver biopsy revealed predominant infiltration of the liver by EBV-infected CD8+ T cells. Liver transplant was conducted as the definitive treatment; however, several days later, relapse of EBV-hepatitis was observed in the donor liver. Although extra-hepatic involvement was scarce, chemotherapy and subsequent bone marrow transplant were attempted in accordance with EBV-LPD. The patient’s liver was free of EBV 2 months after the bone marrow transplant. Our report is the first to imply that acute liver failure caused by EBV-infected T cells might require HCT as the definitive therapy in addition to liver transplantation.

## Case Report

**Figure 1:**
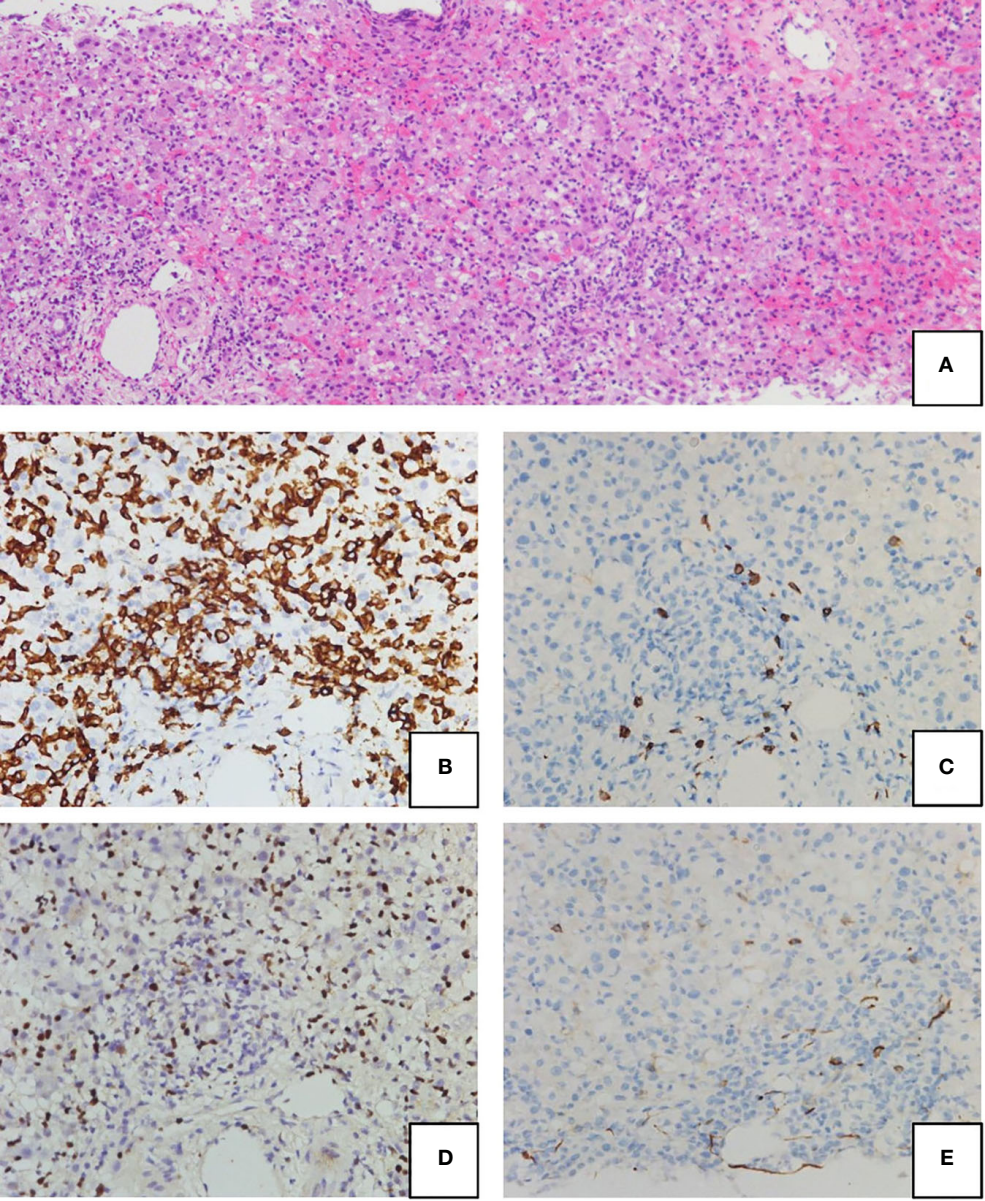
Hematoxylin-eosin (H&E) staining **(A)** and immunohistochemical staining **(B–E)** of the liver biopsy at presentation. Portal lymphocyte infiltration and subsequent massive necrosis were observed **(A)**. Lymphocytes were stained positive for CD8 **(B)** and EBV-encoded small RNA (EBER) **(C)**, but were negative for CD20 **(D)** and CD56 **(E)**.

A previously healthy 2-year-old boy was referred to our hospital with a 2-day history of fever, jaundice, and abdominal pain. Blood tests revealed elevated transaminases (AST, 8,988 IU/L; ALT, 7,480 IU/L), total-bilirubin (6.9 mg/dL), direct-bilirubin (5.6mg/dL), and a prolonged PT-INR of 3.77, all of which were compatible with pediatric acute liver failure (ALF). In addition, a hyperinflammatory state was suggested by sIL-2R (6,970 U/mL) and ferritin (3,434 ng/mL). Liver biopsy revealed fulminant hepatitis, with portal lymphocyte infiltration and massive necrosis (**Figure 1A**). The infiltrating lymphocytes stained positive for CD8 and EBER, but not for CD20 and CD56 (**Figures 1B**–**E**). Therefore, liver failure was thought to be caused by EBV-infected CD8+ T cells. Of note, the results of EBV-specific antibody tests were VCA-IgG+, VCA-IgM-, EBNA+, suggesting past infection. Intensive care, including steroid pulse therapy, plasmapheresis, and continuous hemodiafiltration, did not improve liver function. Therefore, on Day 9 post-admission, the patient received an urgent living donor liver transplant using a left lobe graft from his ABO-identical mother. The amount of EBV-DNA in the explanted liver was 340,000 copies/µg DNA, which was extremely high compared with that in the peripheral blood (2,100 copies/µg DNA).

**Figure 2:**
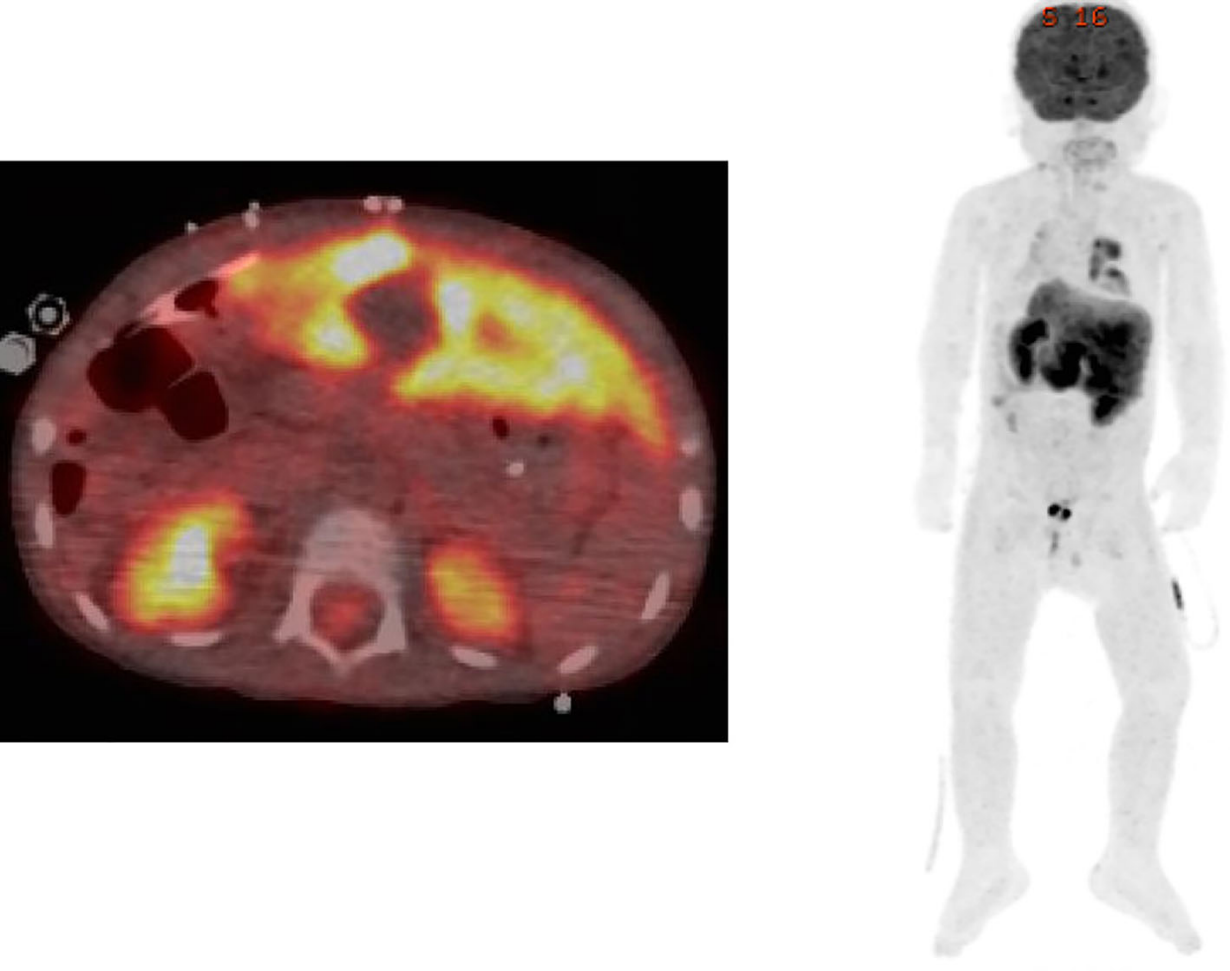
FDG-PET/CT image showing that abnormal uptake is confined to the liver.

**Figure 3:**
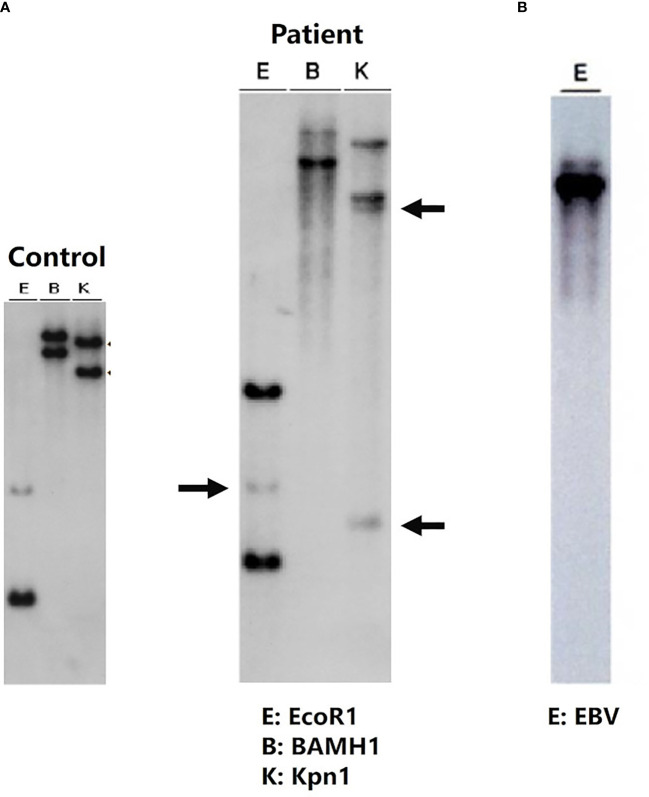
Clonality analysis: clonal rearrangement of the T-cell receptor γ gene **(A)**. Southern blot analysis of the EB viral terminal repeat fragment **(B)** shows monoclonal proliferation of EB virus-infected cytotoxic T lymphocytes. Southern blot analysis of liver samples with the J gamma probe detected rearranged DNA fragments after digestion with EcoR1, BamH1, and Kpn-1 (middle blot). Rearrangement bands are indicated by arrows.

Although the postoperative course seemed uneventful, transaminase levels began to increase on postoperative Day (POD) 5. Follow-up liver biopsy on POD 10 revealed re-infiltration by EBER-positive CD8^+^ T cells, along with portal inflammation and endotheliitis, which are compatible with acute cellular rejection. FDG-PET/CT did not reveal any systemic lesions, and abnormal uptake was confined to the liver (**Figure 2**). Southern blot analysis of the viral terminal repeat fragment identified clonal proliferation of EBV, which along with clonal rearrangement of the T-cell receptor γ gene indicated monoclonal proliferation of EBV-infected CD8^+^ T cells (**Figure 3**).

These results suggest a pathogenesis reminiscent of EBV-HLH; therefore, a chemotherapeutic regimen that included dexamethasone and etoposide was prescribed in accordance with the HLH-2004 protocol (2). Anti-thymocyte globulin was added to control both EBV-HLH and liver allograft rejection. This combination therapy improved liver function. Maintenance treatment with etoposide (50 mg/m^2^ twice per week) led to sustained normalization of liver function and a gradual decrease in the EBV viral load in the peripheral blood and liver. However, the attempt to extend etoposide infusion intervals from POD 80 caused the EBV viral load and transaminase levels to increase. The patient was considered refractory to chemotherapy and underwent HCT from a HLA-full matched unrelated donor with reduced-intensity conditioning (fludarabine, 30 mg/m^2^ from Day -7 to -2; anti-thymocyte globulin, 1.25 mg/kg on Day -7 and -6; melphalan, 60 mg/m^2^ on Day -3 and -2; etoposide, 100mg/m^2^ on Day -3 and -2; and total body irradiation, 3 Gy/1fr on Day -1) (3). Of note, the patient’s liver had two allele mismatches with the bone marrow of the HCT donor.

**Figure 4:**
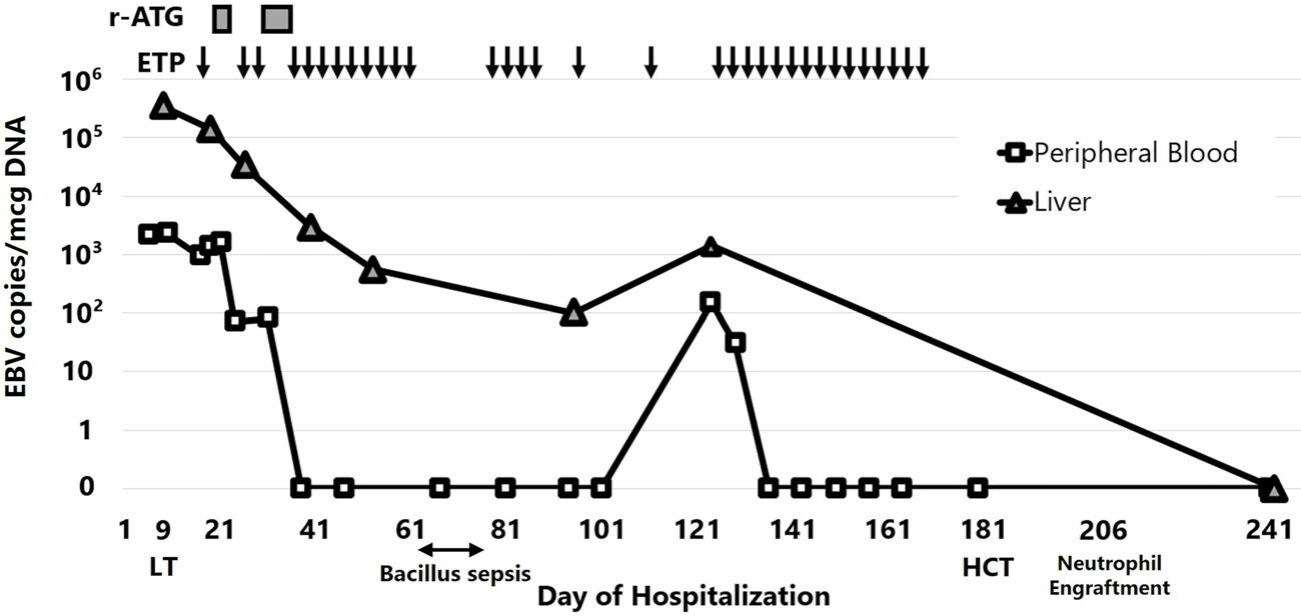
Clinical course of the patient. Periodic administration of etoposide successfully reduced the EBV viral load in the peripheral blood and liver, but recurrence occurred after extending the intervals. r-ATG, rabbit anti-thymocyte globulin; ETP, etoposide; LT, liver transplantation; HCT, hematopoietic cell transplantation.

The patient achieved neutrophil engraftment 25 days after HCT. Liver biopsy performed 2 months after HCT revealed complete eradication of EBV. Later, trio-based whole-exome sequencing did not identify variant genes responsible for familial HLH or congenital susceptibility to EBV infection. The patient continues to do well, with no signs of liver damage (**Figure 4**).

## Discussion

EBV infection is a relatively common cause of viral-induced liver injury, and accounts for 1.1% of pediatric ALF cases (4). By contrast, EBV infection is associated with one-third of HLH cases (5). Indeed, our case met five of the HLH-2004 criteria: fever, low fibrinogen (102 mg/dL), high ferritin (3,434 ng/mL), high soluble IL-2 receptor (6,970 U/mL), and cytopenia (anemia and thrombocytopenia). However, these laboratory data were thought initially to be the presenting features of ALF. In general, EBV-induced liver injury is caused by the T lymphocyte-driven immune response against EBV-infected B cells, although severe cases can be caused by EBV-infected CD8+ T cells (6). In the end, the necessity of chemotherapy and HCT, even after liver transplantation, made our patient’s clinical course clearly distinct from that of typical self-limiting EBV-associated liver damage.

In our case, liver biopsy revealed that ALF was caused by the monoclonal expansion and massive infiltration of EBV-infected CD8+ T cells. Therefore, a diagnosis of an EBV-lymphoproliferative disorders such as EBV-HLH or CAEBV was considered. At initial presentation, the acute deterioration and lack of precedent infectious mononucleosis-like symptoms favoured EBV-HLH over CAEBV. However, the patient lacked overt signs of systemic involvement, and the peripheral EBV viral load was much lower than that of typical EBV-HLH, which generally exceeds 10⁶ copies/µg DNA (7), with clear contrast to the extremely high EBV viral load in the explanted liver. The liver-restricted inflammatory pathology and the entire clinical course, ultimately requiring HCT to cure the disease, favoured CAEBV over EBV-HLH. One explanation for liver-predominant infiltration by EBV-infected CD8+ T cells would be that these cells were trapped in the liver by ICAM-1 expressed on sinusoidal endothelial cells (8). Another possibility is that EBV infection and subsequent clonal proliferation might have occurred in a specific and highly hepatotropic T cell compartment, such as tissue-resident memory T cells (9).

To the best of our knowledge, there are seven case reports of EBV-induced pediatric ALF that required liver transplantation (10–14). Interestingly, in four cases, liver biopsy revealed the infiltrating cells to be EBV-infected CD8+ T cells. Our case, and cases from the past reports followed a similar hyper-acute clinical course until liver transplantation, likely constituting a clinical entity, although our case was the first to necessitate subsequent HCT. Fitting this clinical entity into an existing disease nomenclature is difficult because the clinical courses that these cases followed are not typical of either EBV-HLH or CAEBV. In general, CAEBV is considered to be a slowly progressive disease with neoplastic features. CAEBV has a poor prognosis and almost always requires HCT. By contrast, EBV-HLH is often defined as an EBV-positive hyperinflammatory condition fulfilling the HLH-2004 criteria and following an acute clinical course. Generally, EBV-HLH has a good prognosis, but 10% of cases require HCT (15). EBV-HLH and CAEBV are relatively and arbitrarily defined nomenclatures for a disease condition, and it is often difficult to draw a strict border between the two. For the current case, with an entire clinical course that finally required HCT, a diagnosis of CAEBV may be appropriate. By contrast, for the past cases that achieved clinical remission by liver transplantation, a diagnosis of EBV-HLH may be more suitable.

In conclusion, this case emphasizes the importance of recognizing the existence of a disease spectrum, in which monoclonal infiltration by EBV-infected hepatotropic CD8+ T cells causes liver-restricted hyperinflammation; this is because there is a possibility of relapse post-liver transplantation. Acute cellular rejection, which is a common cause of liver injury after liver transplantation, requires high-intensity immunosuppressive treatment. However, our case required chemotherapy and HCT. Hence in severe EBV-associated ALF, identification of the cell type infected by EBV, and clonality analysis, would be important for an accurate diagnosis and for providing appropriate treatment to patients.

## Data Availability Statement

The original contributions presented in the study are included in the article. Further inquiries can be directed to the corresponding author.

## Ethics Statement

Written informed consent was obtained from the minor(s)’ legal guardian/next of kin for the publication of any potentially identifiable images or data included in this article.

## Author Contributions

KN wrote the case report and prepared the figures. EH was the attending physician of this patient and the director of the whole writing process. HN, KK, YH, KI, and TY performed the diagnostic evaluation of the patient. EO, MS, TO, and HO performed liver transplant surgery. NK, IK, and JT were responsible for the bone marrow transplant procedure. All authors contributed to the article and approved the manuscript.

## Conflict of Interest

The authors declare that the research was conducted in the absence of any commercial or financial relationships that could be construed as a potential conflict of interest.

## Publisher’s Note

All claims expressed in this article are solely those of the authors and do not necessarily represent those of their affiliated organizations, or those of the publisher, the editors and the reviewers. Any product that may be evaluated in this article, or claim that may be made by its manufacturer, is not guaranteed or endorsed by the publisher.
